# Enhancing Consumer Acceptance of Cricket (*Acheta domesticus*) Protein Hydrolysate Through ProteAX‐Mediated Enzymatic Hydrolysis to Improve Techno‐Functional and Organoleptic Properties

**DOI:** 10.1002/fsn3.72033

**Published:** 2026-06-17

**Authors:** Fetriyuna Fetriyuna, Rozanah Nushrotina, Ade Chandra Iwansyah, Nandi Sukri, Ratna Chrismiari Purwestri

**Affiliations:** ^1^ Department of Food Technology, Faculty of Agro‐Industrial Technology Universitas Padjadjaran Jatinangor Indonesia; ^2^ Padjadjaran Center for Sweet Potato Research and Innovation Excellence (PRAISE) Jatinangor Indonesia; ^3^ Research Center for Food Technology and Processing National Research and Innovation Agency Gading Indonesia; ^4^ Faculty and Forestry and Wood Sciences Czech University of Life Sciences Prague Praha‐Suchdol the Czech Republic

**Keywords:** cricket, enzymatic hydrolysis, ProteAX enzyme, protein

## Abstract

Cricket ( *Acheta domesticus* ) protein offers a sustainable solution to Indonesia's malnutrition and food security challenges, stemming from low animal protein consumption and the limited sustainability of conventional livestock systems. Despite its potential as a future protein source, cricket consumption acceptance remains low. This study aimed to investigate the impact of ProteAX enzyme concentration (E/S) and hydrolysis duration on the techno‐functional and organoleptic characteristics of cricket protein hydrolysate flour. The experimental method employed a Factorial Completely Randomized Design with ProteAX enzyme concentrations (0.5%, 1.0%, 1.5%, and 2%) and hydrolysis times (2.0, 3.5, and 5.0 h) factors. Functional properties (degree of hydrolysis, protein content, solubility, emulsifying capacity, total solids and water absorption capacity as well as oil absorption capacity) were determined. The taste, bitter aftertaste and umami organoleptic evaluation were also made. Based on the degree of hydrolysis, techno‐functional and sensory attributes, hydrolysate was achieved at 1.5% enzyme concentration for 5 h, resulting in 67.71% hydrolysis degree, low bitterness intensity (5.9 mm) and aftertaste (2.8 mm), and strong umami flavor (52.16 mm). The statistical analysis shows that enzyme concentration and hydrolysis time independently affect degree of hydrolysis and bitter taste, with no significant interaction, while aftertaste and umami taste show an interaction. This hydrolysate showed increased protein content (69.51%) and improved functional properties, including enhanced solubility (98.99%), water absorption (370.46%), oil absorption (249.35%), and emulsion stability across different pH conditions. These findings support the development of more acceptable insect‐based food products.

## Graphical abstract

ProteAX‐assisted hydrolysis of cricket ( *Acheta domesticus* ) protein enhanced its techno‐functional and sensory properties. Optimal hydrolysis at a 1.5% enzyme concentration for 5 h produced a hydrolysate with a 67.71% degree of hydrolysis, 69.51% protein content, high solubility, improved water and oil absorption capacities, low bitterness, and a strong umami taste, demonstrating its potential as a sustainable food protein ingredient.

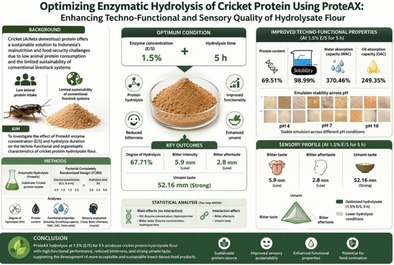

## 1 Introduction

Indonesia faces a serious challenge in food security, with approximately 8.53% of the population (±23.9 million people) experiencing food insufficiency and a projected population growth of 22.05% by 2050 (Badan Pusat Statistik 2023a). Indonesia has the greatest number of malnourished individuals in Southeast Asia, around 17.7 million (Badan Pusat Statistik 2023a, 2023b; Andaruisworo 2021). It is primarily due to imbalanced diet composition (low animal protein consumption), the high cost of livestock production, and a domestic protein shortage (cattle and buffalo) estimated at 374.1 thousand tons resulting in dependency on imports. Moreover, traditional livestock production emits about 18% of the world's greenhouse gases and uses vast land and water resources, calling for more sustainable alternative sources of protein to bolster food security (Govorushko 2019; Baiano 2020; Steinfeld et al. 2006).

Edible insects, especially crickets ( *Acheta domesticus* ), are an increasingly viable source of sustainable protein. Compared to several other edible insects, as well as conventional livestock products (e.g., beef, chicken, and eggs) and legumes (e.g., soybeans and lentils), crickets have higher protein level (Ramos‐Elorduy et al. 2012; Rumpold and Schlüter 2015; Bbosa et al. 2019; Liceaga et al. 2022; Latunde‐Dada et al. 2016). Additionally, crickets have an advantageous profile of the essential amino acids leucine and methionine as well as being higher in essential minerals (iron, magnesium and calcium) than many conventional animal protein sources. From an ecological point of view, cricket has a very low greenhouse gas footprint as it contributes only 1% of the contribution made by ruminant livestock per body weight while at the same time taking up much lesser land and water resources (Baiano 2020; Moruzzo et al. 2021).

In contrast, cricket‐based goods are less accepted by consumers due to undesirable sensory properties and restricted techno‐functional performance (bitter and astringent flavor, poor protein solubility and feeble emulsifying capability). An appropriate approach to overcome such limitations is enzymatic hydrolysis using a mixture of endopeptidase and exopeptidase enzymes towards the reduction of bitter peptides and enhancement of protein functionality. ProteAX enzyme is characterized by endopeptidase and exopeptidase properties, with an optimal temperature of around 60°C and pH 7. The optimal enzyme‐to‐substrate ratio and hydrolysis duration for modifying cricket protein have not been extensively studied. Thus, additional studies are required to assess their impact on the techno‐functional and organoleptic properties of produced protein hydrolysates (Xia et al. 2022; Luna et al. 2021; Cheung et al. 2015; Liu et al. 2016; Witono 2013; Kim et al. 2011; Westemeyer and Dietsch 2023).

## 2 Materials and Methods

### 2.1 Materials

The materials used to prepare the hydrolysate were ProteAX enzyme (activity 1.620 U/g, Amano Enzyme Inc., Nishiki, Japan) and fresh crickets ( *Acheta domesticus* ) obtained from a cricket farm (Rancaekek, Indonesia) with PUR 511 as a feed. The materials used for testing were distilled water, alcohol, bovine serum albumin (BSA), CuSO_4_ (copper (II) sulfate), Folin Ciocalteu, H_2_SO_4_ (sulfuric acid), HCl (hydrochloric acid), KNaC_4_H_4_O_6_·4H_2_O (potassium sodium tartrate), Na_2_CO_3_ (sodium carbonate), NaOH (sodium hydroxide), and TCA (trichloroacetic acid).

### 2.2 Methods

This study used an experimental method with descriptive data analysis. The experimental design was a Factorial Completely Randomized Design (FCRD), combining two variables: ProteAX enzyme concentration (0.5%, 1%, 1.5%, and 2% w/v) and hydrolysis time (2, 3.5, and 5 h). This resulted in 12 different treatments, plus a control without enzyme addition. Each treatment was repeated 3 times. Data were analyzed using ANOVA, followed by Duncan's test if significant differences were found.

#### 2.2.1 Preparation of Samples

Cricket mash (CM) was made by blending whole crickets into a homogeneous paste. Cricket protein (CP) was obtained after sample preparation and freeze‐drying without enzymatic treatment. The hydrolysis conditions of the systems were established, and then enzymatic‐treated cricket protein was used to produce cricket protein hydrolysate (CPH) by ProteAX enzyme.

Cricket hydrolysate was modified from previous research (Hilkias et al. 2017). Frozen crickets were separated from their caput and antennae, then thawed at 4°C. They were rinsed, mixed with distilled water at a 1:2 (w/v) ratio, and blended for 2 min (CM). The mixture was pasteurized in a water bath at 90°C for 15 min. Pasteurized samples were pre‐incubated at pH 7.0 and 60°C, the optimal temperature for ProteAX enzyme activity, with pH adjustments made using 5 M NaOH. Samples were treated with ProteAX enzyme at concentrations of 0%, 0.5%, 1.0%, 1.5% and 2% for 2, 3.5, and 5 h. Hydrolysis occurred in a shaking incubator at 100 rpm. After hydrolysis, the enzyme was inactivated at 90°C for 20 min in a water bath. The hydrolysate was centrifuged at 8000 *g* for 20 min at 5°C, and the supernatant was freeze‐dried at −85°C for ±60 h to obtain cricket protein hydrolysate.

#### 2.2.2 Organoleptic Test

Organoleptic testing was conducted to assess umami, bitterness, and aftertaste of cricket protein hydrolysate using the Generalized Labeled Magnitude Scale (gLMS) on a 150 mm line with 6 scales used were barely detectable (2.1 mm), weak (9 mm), moderate (25.5 mm), strong (52.05 mm), very strong (78.75 mm), and strongest imaginable sensation (150 mm) (Morais et al. 2013). Panelists consisted of 25 semi‐trained individuals who received prior instructions on the sensory evaluation procedures before the test. Participants were selected based on the following inclusion criteria: being in good health, having no allergies to grasshoppers or related products, being able to read and write, and being non‐smokers. Individuals were excluded if they had allergies to grasshoppers, shrimp, or similar products, were in poor health during the evaluation, were smokers, were unable to read and write, or were younger than 16 years or older than 25 years. These criteria were applied to ensure reliable sensory assessments. Panelists held the sample on their tongue for 10 s, then spat it out. The taste was immediately evaluated with gLMS, followed by a 1–2 s rinse. After 5 min, the aftertaste was assessed using the same scale. The sensory evaluation protocol was approved by the Research Ethics Committee of Universitas Padjadjaran, Indonesia (Approval No. 9/UN6.KEP/EC/2025).

#### 2.2.3 Hydrolysis Degree Test

The degree of hydrolysis (DH) was determined using the trichloroacetic acid (TCA)‐soluble nitrogen method with slight modification from a previous study (Lowry et al. 1951). A 5 mL aliquot of hydrolysate supernatant was pipetted and mixed with 5 mL of 20% TCA. The sample was left to stand for 30 min. Next, the sample was centrifuged at 8000 *g* for 20 min. The nitrogen content of the resulting supernatant and hydrolyzed sample was then analyzed using the Lowry method (Adler‐Nissen 1979). The degree of hydrolysis can be calculated using the following formula:

$$\mathrm{DH}=\frac{\text{Soluble proteins}}{\text{Total protein}}\times 100$$

## 3 Result and Discussion

### 3.1 Evaluation of the Enzymatic Hydrolysis Process

#### 3.1.1 Evaluation of Degree of Hydrolysis (DH) Value

The DH value reflects the percentage of peptide bonds hydrolyzed during the process. This method is effective for assessing how much protein is broken down into smaller peptides and amino acids, serving as an indicator of the efficiency of the hydrolysis process (Bhaskar et al. 2008). The results of the hydrolysis degree test for cricket protein hydrolysate are shown in the following Table 1.

**TABLE 1:** Hydrolysis degree testing.

Sample	Enzyme concentration (E/S) (%)	Time (h)	DH (%)
I	0.5	2	50.34 ± 1.88
II	1.0	2	51.61 ± 1.81
III	1.5	2	53.03 ± 1.40
IV	2	2	53.40 ± 1.80
V	0.5	3.5	55.13 ± 2.18
VI	1.0	3.5	56.25 ± 1.80
VII	1.5	3.5	59.58 ± 2.27
VIII	2	3.5	58.38 ± 1.65
IX	0.5	5	63.12 ± 2.52
X	1.0	5	64.69 ± 0.88
XI	1.5	5	67.71 ± 2.22
XII	2	5	66.75 ± 1.61
CP	0	0	6.85 ± 0.56

*Note:* All data are presented as mean ± standard deviation. Discrepancies between predicted and actual results may be due to environmental factors affecting the conditions during laboratory testing. Samples I–XII are coded samples differentiated based on enzyme concentration (E/S) treatments and hydrolysis duration, as outlined in the corresponding table *F* = degree of variations explained by a factor compared to random values, *p*‐value = significant level at *p* < 0.05.

Table 1 shows that the Degree of Hydrolysis (DH) for the raw cricket protein (CP) sample is 6.85%, indicating the presence of large protein or peptide molecules still soluble in TCA. Meanwhile, the hydrolyzed samples exhibited DH values ranging from 50.34% to 67.71%, indicating the successful completion of the hydrolysis process as it increased DH values to exceed 50%.

Based on the results of ANOVA analysis, both the E/S and hydrolysis duration exhibited significant individual effects on DH value (*F*_E/S_ = 8.809, df_E/S_ = 3, *p*‐value = < 0.01; *F*_*D*_ = 166.65, df_*D*_ = 2, *p*‐value = < 0.01; *p* < 0.05), but no significant for interaction was observed between these two factors (*F*_interaction_ = 0.228, df = 6, *p*_interaction_‐value = 0.964, *p* > 0.05). This independence indicates that the impact of enzyme concentration on DH remains consistent regardless of hydrolysis duration, and similarly, the effect of hydrolysis duration remains constant across different enzyme concentrations. Since these factors operate independently without meaningful synergistic effects on DH values. The relationship between enzyme concentration and DH values demonstrates a consistent pattern regardless of the hydrolysis duration. This means that changes in enzyme concentration will produce similar effects on DH values whether the hydrolysis is conducted for a short or long period. Conversely, the impact of hydrolysis duration remains constant across different enzyme concentration levels, indicating that the time‐dependent effects on protein breakdown are not significantly altered by varying enzyme concentrations. This independence between factors suggests that the enzymatic hydrolysis process follows predictable patterns where the influence of enzyme concentration remains stable over time, and the effects of hydrolysis duration maintain their consistency regardless of how much enzyme is present in the reaction mixture.

#### 3.1.2 Effect of Enzyme Concentration (E/S) on DH Value

To determine the best enzyme concentration (E/S) factor, Duncan's statistical test was performed by grouping the mean DH values based on their significance levels. The following are the results obtained (Table 2).

**TABLE 2:** Correlation between enzyme concentration (E/S) and DH values (*n* = 12).

Enzyme concentration (E/S)	DH values (%)
0.5%	56.19^a^
1%	57.53^a^
1.5%	60.11^b^
2%	59.51^b^
*F*‐value	8.809
df	3
*p*‐value	< 0.05

*Note:* Data were analyzed using Duncan's multiple range test; Different superscript letters indicate significant differences (*p* < 0.05).

The results indicate that higher enzyme concentrations (1.5% and 2%) are highly significant difference compared to lower concentrations (0.5% and 1%). However, within smaller ranges (0.5%–1% or 1.5%–2%), the increase in enzyme concentration did not have a statistically significant impact. An enzyme concentration (E/S) of 1.5% was selected as the optimal conditions, as it achieved the highest DH with more efficient enzyme usage compared to 2%. This aligns with other research showing DH values reaching optimal levels at 1.5% enzyme concentration before entering a stationary phase (Bahri et al. 2021). The decrease in DH at 2% E/S is likely due to suboptimal environmental conditions affecting oligopeptide and amino acid production, enzyme aggregation reducing substrate binding efficiency, and certain hydrolysis byproducts inhibiting protease activity (Alahmad et al. 2023; Sharikov et al. 2016). The decline in DH can also be attributed to excess enzyme concentration, where reaction rates reach saturation as available enzymes exceed hydrolysable substrate. High enzyme concentrations can lead to enzyme competition for substrate binding, reduced hydrolysis efficiency, and potential enzymatic denaturation. Studies by various researchers demonstrate that excessive enzyme concentrations can impair hydrolysis efficiency due to limited enzyme activity and mass transfer (Zapata‐Montoya et al. 2018; Bisswanger 2014). Enzyme activity shows a pattern where it increases linearly with substrate concentration until reaching a maximum point, after which additional substrate concentration no longer affects enzyme reaction rate, demonstrating saturation kinetics (Mahdavi‐Yekta et al. 2019).

Enzyme concentration plays a crucial role in protein hydrolysis, directly influencing the degree of hydrolysis (DH). Increasing enzyme concentration enables more substrate binding, resulting in greater peptide bond breakdown (You and Zhang 2014). Research emphasizes the significance of enzyme concentration in optimizing enzymatic processes (Muñoz‐Seijas et al. 2024). *Tenebrio molitor* (mealworm) protein hydrolysis using *Aspergillus oryzae* protease showing DH increased from 47% to 78% with enzyme concentration enhancement (Herman et al. 2022). Similarly, protein hydrolysis of *Bombyx mori* (silkworm) pupae, revealing a DH increase up to 97.46%, further supporting the positive correlation between enzyme activity and protein hydrolysis efficiency (Lestari et al. 2020).

#### 3.1.3 Effect of Hydrolysis Duration on DH Values

Hydrolysis duration also plays a significant role in determining DH value. Below are the results of the comparison of mean DH values at different times based on Duncan's statistical test (Table 3).

**TABLE 3:** Correlation between hydrolysis duration and DH values.

Time (h)	DH values (%)
2 h	52.09^a^
3.5 h	57.34^b^
5 h	65.57^c^
*F*‐value	84.64
df	2
*p*‐value	< 0.05

*Note:* Data were analyzed using Duncan's multiple range test; Different superscript letters indicate significant differences (*p* < 0.05).

Longer hydrolysis durations result in higher DH values, with significant increases at each tested time interval. The optimal treatment is a 5‐h hydrolysis, yielding the highest DH value. Hydrolysis duration influences both the quantity of amino acids and the types of peptides produced which impact the DH increase (Alahmad et al. 2023). Previous research demonstrated a linear increase in DH from 13.36% to 20.15% with increasing hydrolysis time in their study of fish protein hydrolysates (Solanki et al. 2023). Similarly, other research revealed a consistent DH increase over time, particularly at higher protein concentrations (Hall et al. 2018).

#### 3.1.4 Effect of Degree of Hydrolysis (DH) Value

High hydrolysis degree (DH) significantly influences various protein characteristics. Previous research demonstrated that hydrolysis increasing DH to 60%–85% can eliminate igE reactivity to tropomyosin, substantially reducing allergenic potential (Trinh and Supawong 2021). High DH values improve functional technical properties, including solubility, foaming capacity, and emulsification characteristics of resulting hydrolysates (Cui et al. 2021). Moreover, the hydrolysis degree serves as a critical indicator for bitterness assessment. Low DH values are a primary contributor to bitter taste in hydrolysates. These findings underscore the importance of precise enzymatic hydrolysis in modifying protein functional properties and sensory characteristics (De Maria et al. 2017).

The increase in DH correlates with enhanced protein unfolding, a critical factor in improving protein degradation (Rajarathnam et al. 2016). This unfolding process enables the breakdown of large molecules into smaller fragments, facilitating protein solubility and digestibility. Protein unfolding influences hydrolysis speed and efficiency by preventing aggregate formation that could inhibit enzyme activity. Previous research demonstrated increased protein content in permeate with rising Degree of Hydrolysis (DH) values (Magara et al. 2021). Consequently, subsequent research focused on the highest DH sample (Sample XI), characterized by 1.5% enzyme concentration over 5 h subsequently labeled as ricket Protein Hydrolysate (CPH). This approach highlights the strategic importance of optimizing enzymatic hydrolysis parameters to enhance protein functionality and digestibility.

### 3.2 Protein Content

Enzymatic hydrolysis is closely related to protein breakdown, as it involves breaking proteins into simpler compounds. When enzymes function optimally, they significantly enhance protein degradation, leading to increased protein fragmentation. In this study, protein content was analyzed across three distinct cricket sample preparations that underwent different hydrolysis processes. The sample variations included: Cricket Mash (CM), which consists of whole crickets blended into a homogeneous mixture; Cricket Protein (CP), representing crickets that underwent a comprehensive preparation process involving initial preparation, incubation, and subsequent freeze‐drying; and Cricket Protein Hydrolysate (CPH), which is a protein hydrolysate derived from crickets with the highest degree of hydrolysis (sample XI) in a dried form. The results of protein content testing in CM, CP, and CPH can be seen in the following table.

Based on Table 4, whole cricket (CM) obtained through blending contains 64.34% protein on a dry basis. This data aligns with another study which reported that crickets contain 55%–73% protein (dry basis) (Chiodza and Goosen 2023). Subsequently, the sample underwent separation of caput and antennae, followed by incubation and drying, resulting in a Cricket Protein (CP) sample. The protein content increased because non‐protein components were reduced, leading to a more concentrated protein sample. The protein content of crickets significantly increased to 79.51% (dry basis) after enzymatic hydrolysis. Enzymatic hydrolysis involves using specific enzymes to break down protein chains, which enhances protein activity and solubility by cleaving peptide bonds. This process increases the accessibility of peptide bonds, thereby improving the extraction of peptides and amino acids. The resulting hydrolysate has a simplified structure that becomes more easily detectable and measurable during protein content analysis. Supporting this approach, other study demonstrated that ProteAX enzymatic hydrolysis can significantly improve protein solubility and increase measurable protein content (Luna et al. 2021). This method represents an effective technique for enhancing protein concentration and accessibility in cricket‐based protein sources.

**TABLE 4:** Cricket protein content.

Sample	Total solid (%)	Protein (WB) (%)	Protein (DB) (%)
CM	27.24 ± 1.16	17.53 ± 0.66	64.34 ± 2.43
CP	89.11 ± 0.03	60.03 ± 0.73	67.37 ± 0.82
CPH	89.56 ± 0.03	71.21 ± 2.76	79.51 ± 2.76

Abbreviations: CM, cricket mash; CP, cricket protein; CPH, cricket protein hydrolysate.

Protease enzyme concentration is crucial for optimizing protein breakdown (Herman et al. 2022). Appropriate enzyme concentration directly enhances protein recovery by releasing soluble proteins through more intensive enzyme‐substrate reactions (Priyanto and Trisna 2022). Generally, increasing enzyme concentration accelerates the time required to reach maximum protein levels (FitzGerald and O'cuinn 2006). However, once the best concentration is reached, enzymes saturate their substrate binding and reaction acceleration capacities. Other studies observed that excessive enzyme concentrations can generate emulsions and precipitates, resulting in a 5%–11% reduction in protein recovery (Priyanto and Trisna 2022). These findings underscore the importance of precise enzyme concentration management in protein hydrolysis processes, highlighting the need for careful optimization to maximize protein extraction and functional characteristics.

### 3.3 Organoleptic Evaluation

Enzymatic hydrolysis breaks down proteins into shorter peptides or free amino acids, which can alter the organoleptic properties of the resulting hydrolysate. Organoleptic testing was performed to evaluate umami, bitterness, and aftertaste of cricket protein hydrolysate using the Generalized Labeled Magnitude Scale (gLMS) on a 150 mm line. The six scales used were barely detectable (2.1 mm), weak (9 mm), moderate (25.5 mm), strong (52.05 mm), very strong (78.75 mm), and the strongest imaginable sensation (150 mm) (Morais et al. 2013). Results are shown in Table 5.

**TABLE 5:** Evaluation of bitter taste based on gLMS values.

Sample	(E/S) (%)	Time (h)	gLMS (mm)
Standard	0	0	54.08 ± 2.48
CP	0	0	32.88 ± 3.32
I	0.5	2	16.16 ± 3.31
II	1.0	2	14.96 ± 2.95
III	1.5	2	12.16 ± 3.82
IV	2	2	13.44 ± 3.08
V	0.5	3.5	12.72 ± 3.45
VI	1.0	3.5	10.56 ± 3.80
VII	1.5	3.5	10.08 ± 3.62
VIII	2	3.5	9.04 ± 2.71
IX	0.5	5	7.84 ± 2.70
X	1.0	5	8.40 ± 2.64
XI	1.5	5	5.92 ± 2.61
XII	2	5	7.12 ± 1.83

*Note:* All data are presented as mean ± standard deviation. Samples I–XII are coded samples differentiated based on enzyme concentration (E/S) treatments and hydrolysis duration, as outlined in the corresponding table. Discrepancies between predicted and actual results may be attributed to the subjective factors of the panelists.

**FIGURE 1:**
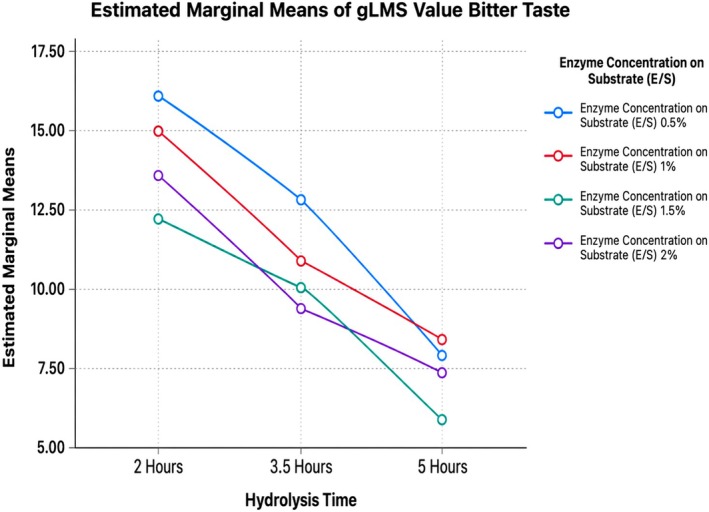
Graph of the effect of enzyme concentration (E/S) on hydrolysis time on bitter taste gLMS values.

The standard gLMS value represents a strong bitter taste, indicating panelists effective detection of bitterness. Native cricket protein (CP) samples showed 32.88 mm, categorized as strong bitterness, while cricket protein hydrolysate samples demonstrated gLMS values ranging from 5.92 to 16.16 mm, indicating successful bitterness reduction from strong to weak categories. Here is the graph showing the effect of enzyme concentration (E/S) on hydrolysis duration and its impact on the gLMS value of bitterness (Figure 1).

Two‐way ANOVA revealed significant effects of enzyme‐to‐substrate ratio (E/S) and hydrolysis time on the gLMS bitterness value (*F*_E/S_ = 13.421, df_E/S_ = 3, *p*‐value = < 0.01; *F*_*D*_ = 122.502, df_*D*_ = 2, *p*‐value = < 0.01, *p* < 0.05), indicating that the two work independently to affect bitterness. However, the interaction between these two factors was not significant (*F*_interaction_ = 1.872, df = 6, *p*_interaction_‐value = 0.086, *p* > 0.05), indicating that each factor influenced bitterness independently. Therefore, enzyme concentration and hydrolysis duration affect the gLMS value independently, without a combined effect, allowing for further analysis of each factor individually. Enzymatic hydrolysis also affects the gLMS aftertaste bitterness value in cricket protein hydrolysates, as shown in the following sensory test results.

**FIGURE 2:**
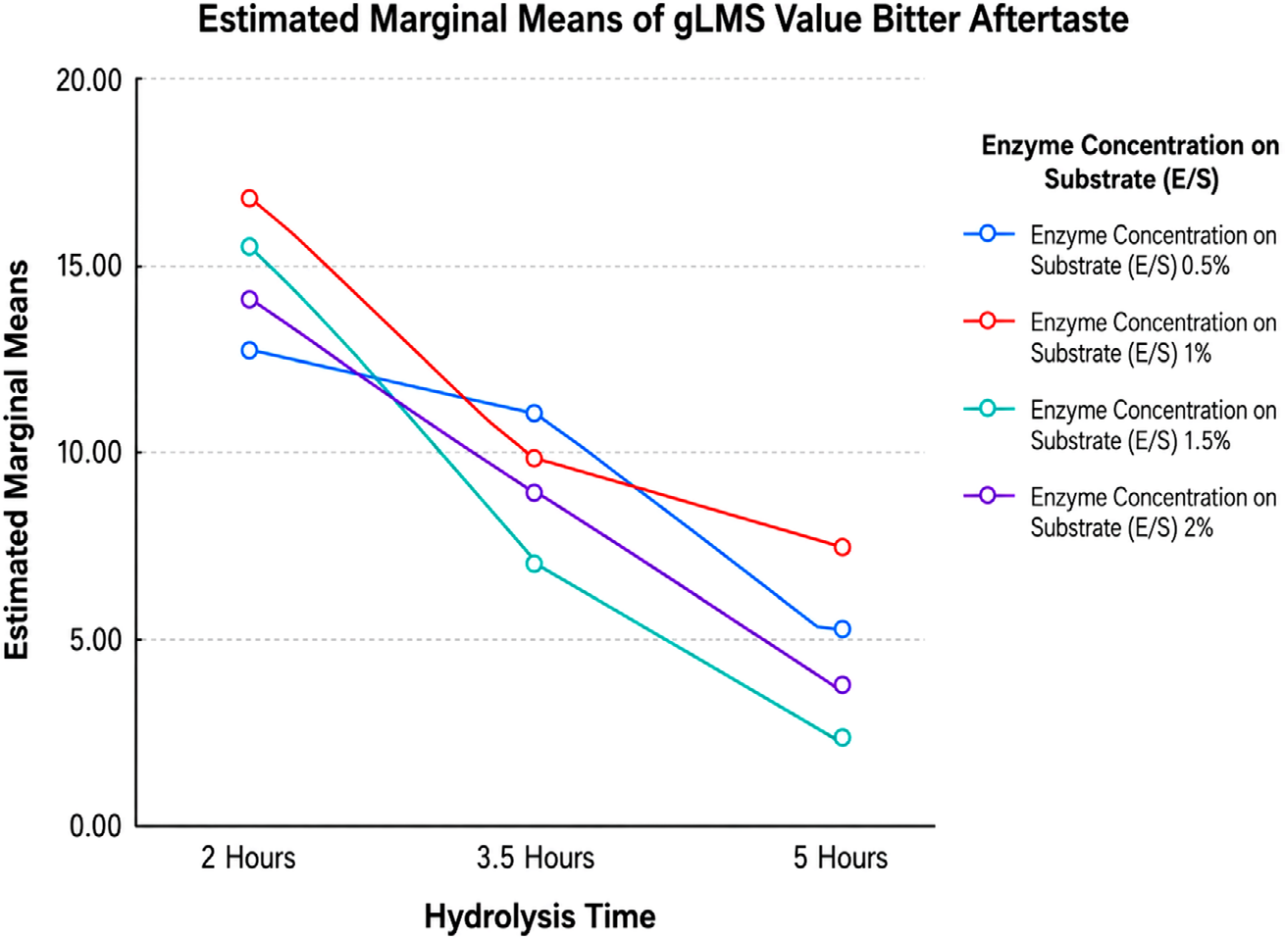
Graph of the effect of enzyme concentration (E/S) on hydrolysis time on bitter aftertaste gLMS values.

Based on Table 6, the standard gLMS value represents a strong bitter aftertaste, indicating panelists effective detection of bitter aftertaste. Additionally, the hydrolysis process successfully reduced bitter aftertaste from a moderate to nearly undetectable level. The following graph shows the effect of enzyme concentration (E/S) on the duration of hydrolysis on the gLMS value of bitter aftertaste (Figure 2).

**TABLE 6:** Evaluation of bitter aftertaste based on gLMS scores.

Sample	Enzym (E/S) (%)	Time (h)	gLMS (mm)
Standard	0	0	52.88 ± 3.37
CP	0	0	20.00 ± 3.32
I	0.5	2	12.72 ± 3.91
II	1.0	2	16.56 ± 3.14
III	1.5	2	15.44 ± 3.94
IV	2	2	13.84 ± 3.51
V	0.5	3.5	10.96 ± 3.70
VI	1.0	3.5	9.84 ± 3.87
VII	1.5	3.5	6.80 ± 3.46
VIII	2	3.5	8.96 ± 3.27
IX	0.5	5	5.28 ± 3.55
X	1.0	5	7.36 ± 3.09
XI	1.5	5	2.80 ± 2.64
XII	2	5	3.92 ± 2.86

*Note:* All data are presented as mean ± standard deviation. Samples I–XII are coded samples differentiated based on enzyme concentration (E/S) treatments and hydrolysis duration, as outlined in the corresponding table. Discrepancies between predicted and actual results may be attributed to the subjective factors of the panelists.

This significant reduction in bitter aftertaste may suggest that enzymatic hydrolysis contributes to modifications in protein characteristics, which could help improve the sensory profile of cricket protein by reducing undesirable taste perceptions. Based on the ANOVA results, both factors and their interaction significantly affected the bitter aftertaste (*F*_interaction_ = 5.362, df = 6, *p*_interaction_‐value = < 0.01, *p* < 0.05), with hydrolysis time having a more dominant influence. At 2 h of hydrolysis, bitter aftertaste varies inconsistently with enzyme concentration. By 3.5 h, a clearer trend emerges where higher enzyme concentrations generally reduce bitter aftertaste. At 5 h, enzyme concentration shows the strongest effect, with a significant decrease in bitter aftertaste.

#### 3.3.1 The Effect of Enzyme Concentration (E/S) on Bitter Taste and Aftertaste

To determine the best enzyme concentration (E/S) factor, Duncan's statistical test was performed by grouping the mean gLMS value based on their significance levels. The following are the obtained results:

Based on Table 7, the results indicate that using a lower enzyme concentration tends to produce a higher bitter taste compared to higher concentrations. An enzyme concentration (E/S) of 1.5% was selected as the best treatment, as it achieved the highest gLMS bitter value with more efficient enzyme usage compared to 2%. Enzyme concentration (E/S) also affects the gLMS bitter aftertaste value in cricket protein hydrolysates, as shown in the correlation analysis below.

**TABLE 7:** Correlation between enzyme concentration (E/S) and gLMS bitter value.

Concentration of enzyme to substrate (E/S)	gLMS (mm)
0.5%	12.24^b^
1%	11.30^b^
1.5%	9.39^a^
2%	9.87^a^
*F*‐value	13.421
df	3
*p*‐value	< 0.05

*Note:* Data were analyzed using Duncan's multiple range test; Different superscript letters indicate significant differences (*p* < 0.05).

Based on Table 8, the enzyme concentration (E/S) of 1.5% is the best treatment as it results in the lowest aftertaste value, although statistically not significantly different from the 2% (E/S) concentration. However, there appears to be no correlation between increasing enzyme concentration and the reduction of gLMS bitter aftertaste value in cricket hydrolysate (based on panelists' evaluations).

**TABLE 8:** Correlation between enzyme concentration (E/S) and gLMS bitter aftertaste value.

Concentration of enzyme to substrate (E/S)	gLMS (mm)
0.5%	9.65^b^
1%	11.25^c^
1.5%	8.35^a^
2%	8.91^ab^
*F*‐value	10.107
df	3
*p*‐value	< 0.05

*Note:* Data were analyzed using Duncan's multiple range test; Different superscript letters indicate significant differences (*p* < 0.05).

Enzymatic hydrolysis primarily works by removing hydrophobic amino acid residues at both peptide ends using N‐terminal or C‐terminal hydrolase to reduce bitterness (Aspevik et al. 2016). Using appropriate proteases and processing conditions, enzymatically hydrolyzed proteins would not produce bitter peptides (Ariyani et al. 2017). The 1.5% enzyme concentration (E/S) proven best, yielding the lowest gLMS values for bitter taste and aftertaste, though a slight, insignificant increase in bitterness occurred at 2% E/S. Other research demonstrated that 1.5% enzyme concentration improved hydrolysis efficiency without dominant bitterness, provided controlled reaction time (Bahri et al. 2021). Protease concentrations above 1.5% increased bitterness due to enhanced short peptide chain formation (Haryati et al. 2010). Increased enzyme levels could reduce bitterness if peptides were broken down into non‐bitter free amino acids (Rosalinda et al. 2021). The increased bitterness at 2% E/S likely resulted from excessive hydrolysis, producing small peptides with hydrophobic groups causing bitter taste and aftertaste (Putri et al. 2020). Additionally, high enzyme concentrations could increase bitterness and bitter aftertaste due to the production of bitter‐causing peptides (Liu et al. 2022).

#### 3.3.2 The Effect of Hydrolysis Time on Taste and Bitter Aftertaste

Based on the ANOVA analysis, the hydrolysis time factor significantly affects the gLMS taste and bitter aftertaste values (*p*‐value < 0.05). Below is the table showing the correlation analysis between the two.

Table 9 shows that as the hydrolysis time increases, the bitter taste and aftertaste value decreases, with a significant reduction at each tested time interval. The best treatment is the 5‐h hydrolysis, as it results in the lowest bitter taste and aftertaste value. There is a consistent pattern between bitter taste and bitter aftertaste, whereas the hydrolysis time increases, the gLMS taste and bitter aftertaste values decrease significantly. This suggests that the components responsible for the initial bitter taste are likely also contributing to the bitter aftertaste. Longer hydrolysis helps break down these components, resulting in a product with a more neutral taste and better aftertaste. Therefore, the 5‐h hydrolysis provides the best results in reducing the intensity of both the bitter taste and bitter aftertaste. The bitterness‐reducing effect of exopeptidase activity becomes more pronounced with longer hydrolysis time (Liu et al. 2016). During the initial hydrolysis stages, the endopeptidase activity of ProteAX breaks peptide bonds within the protein chain through non‐thermal processes. This disrupts the compact protein structure, consequently releasing bitter peptides (Ariyani et al. 2017). Subsequently, structural changes occur in these bitter peptides.

**TABLE 9:** Correlation between hydrolysis duration and gLMS bitter taste and aftertaste value.

Time (h)	gLMS bitter taste (mm)	gLMS bitter aftertaste (mm)
2	14.18^c^	14.64^c^
3.5	10.60^b^	9.14^b^
5	7.32^a^	4.84^a^

*Note:* Data were analyzed using Duncan's multiple range test; Different superscript letters indicate significant differences (*p* < 0.05).

#### 3.3.3 The Effect of Degree of Hydrolysis on Taste and Bitter Aftertaste

The bitterness of hydrolysates is closely associated with DH value and the type of protease used during the hydrolysis process (Newman et al. 2014). This research reveals that high bitter taste and bitter aftertaste are predominantly observed in hydrolysates with lower DH values. According to other research, bitterness intensity tends to be higher at extremely low DH levels (Nakamura et al. 2023). The reduction in bitterness can be attributed to the role of ProteAX enzyme, which possesses aminopeptidase activity. This enzymatic activity effectively releases N‐terminal amino acids from peptides, consequently mitigating bitterness. Furthermore, ProteAX may exhibit relatively weak endopeptidase activity, which could contribute to the production of fewer bitter peptides (Wei et al. 2024).

According to other research, hydrolysates with DH of 16% exhibit increased Surface Hydrophobicity (SH), resulting in a more intense bitter taste, particularly for molecules with molecular weights between 500 and 1000 Da and high hydrophobicity (Xiang et al. 2024). As the DH increases from 16% to 20%, the surface hydrophobicity of hydrolysates decreases. This reduction occurs because larger peptides are progressively broken down into smaller fragments, leading to a diminished hydrophobic area. When the DH reaches 20%, hydrophobic exposure begins to decline, causing changes in bitter taste perception (Xiang et al. 2024). Other research demonstrates that when the degree of hydrolysis increases to 20%, ProteAX exhibits strong exopeptidase activity, effectively breaking down peptides at the protein chain ends (Xiang et al. 2024; Witono et al. 2007). The enzyme can cleave numerous hydrophobic amino acids (HAA) from the C‐terminal of bitter peptides, significantly reducing the proportion of HAA in hydrolysate samples and consequently mitigating bitterness. The exopeptidase function of ProteAX further degrades bitter peptides, releasing smaller peptides and free amino acids (Liu et al. 2016; Nakamura et al. 2023).

#### 3.3.4 Evaluation of gLMS Umami Taste Values

Hydrolysis also can transform proteins into l‐amino acids, nucleotides, and various peptides that can impart umami taste to foods (Prayudi et al. 2019).

**FIGURE 3:**
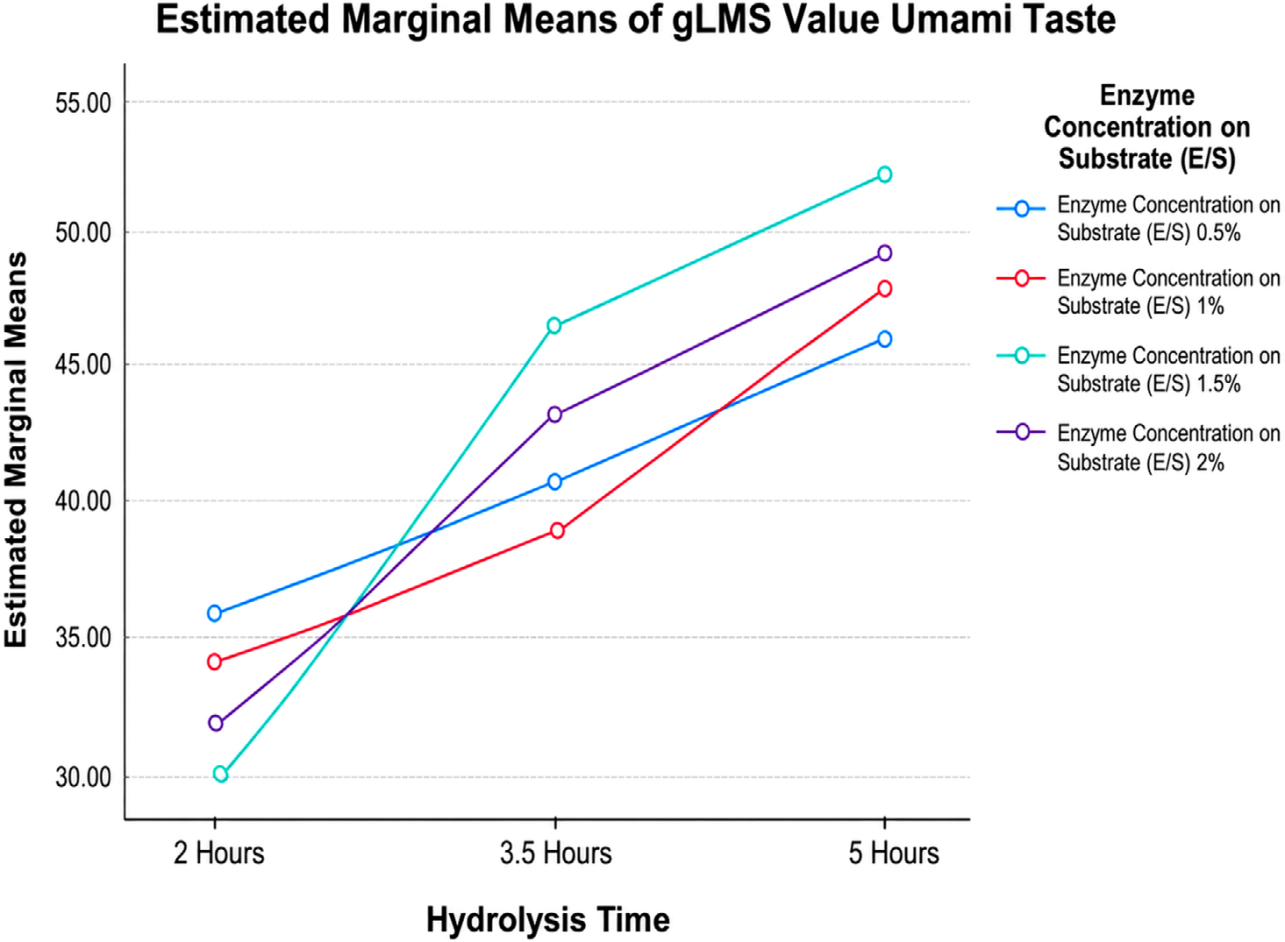
Graph of the effect of enzyme concentration (E/S) on hydrolysis time on umami taste gLMS values.

Based on Table 10 and Figure 3, enzymatic hydrolysis significantly enhanced umami taste in cricket protein samples. Sample XI demonstrated the strongest umami flavor (52.16 mm), while Sample III represented the lowest intensity (30.32 mm). Based on ANOVA analysis, both enzyme concentration and hydrolysis time had a significant effect on umami taste (*F*_interaction_ = 21.111, df = 6, *p*_interaction_‐value = < 0.01, *p* < 0.05). A strong interaction between enzyme concentration and hydrolysis time was also detected, showing that the effect of enzyme concentration depended on the hydrolysis time. In particular, the results of ANOVA showed that hydrolysis time was a stronger contributor to variation in umami taste than enzyme concentration and the interaction term also showed a significant effect.

**TABLE 10:** Evaluation of umami taste based on gLMS value.

Sample	(E/S) (%)	Time (h)	gLMS (mm)
CP	0	0	20.40 ± 3.92
I	0.5	2	35.84 ± 2.08
II	1.0	2	34.32 ± 2.14
III	1.5	2	30.32 ± 3.20
IV	2	2	32.08 ± 3.58
V	0.5	3.5	40.8 ± 3.74
VI	1.0	3.5	39.08 ± 2.77
VII	1.5	3.5	46.56 ± 4.26
VIII	2	3.5	43.44 ± 3.54
IX	0.5	5	46.08 ± 3.67
X	1.0	5	47.84 ± 3.95
XI	1.5	5	52.16 ± 3.60
XII	2	5	49.20 ± 3.32

*Note:* All data are presented as mean ± standard deviation. Samples I–XII are coded samples differentiated based on enzyme concentration (E/S) treatments and hydrolysis duration, as outlined in the corresponding table. Discrepancies between predicted and actual results may be attributed to the subjective factors of the panelists.

#### 3.3.5 Effect of Enzyme Concentration (E/S) on Umami Taste

To determine the best enzyme concentration (E/S) with higher umami taste, duncan's statistical test was performed by grouping the mean gLMS value based on their significance levels. The following are the obtained results.

Based on Table 11, enzyme concentration (E/S) of 1% produced the lowest umami taste score, followed by concentrations of 0.5% and 2% with similar scores, while concentration 1.5% achieved the highest score, significantly different from other treatment. Thus, 1.5% is the best concentration for maximizing umami taste. Protease enzymes play a key role in enhancing protein hydrolysis into amino acids and umami‐forming peptides. Previous research showed that increasing enzyme concentration can optimize hydrolysis, leading to enhanced umami taste (Parhusip et al. 2024). This aligns with other research finding that higher protease enzyme concentrations produce elevated glutamic acid content, intensifying umami flavor in hydrolysates (Wicaksono and Winarti 2021). Study on endopeptidase and exopeptidase enzyme mixture comparisons demonstrated that optimal enzyme concentration could produce the best umami taste through melanoidin and amino acid formation (Rosida 2022). Additionally, other research found that a 1.5% enzyme concentration produced optimal compound profiles contributing to savory and umami flavors (Gunadi 2021). Similarly study on catfish hydrolysis using protease at 1.5% enzyme concentration showed that it produced dominant short peptides resulting in umami taste (Fajriyah and Winarti 2022).

**TABLE 11:** Correlation between enzyme concentration (E/S) and gLMS umami taste value.

Concentration of enzyme to substrate (E/S)	gLMS (mm)
0.5%	40.91^ab^
1%	40.41^a^
1.5%	43.01^c^
2%	41.57^b^
*F*‐value	8.356
df	3
*p*‐value	< 0.05

*Note:* Data were analyzed using Duncan's multiple range test; Different superscript letters indicate significant differences (*p* < 0.05).

#### 3.3.6 Effect of Hydrolysis Time on Umami Taste

Based on the ANOVA analysis, the hydrolysis time factor significantly affects the gLMS umami taste (*p* < 0.05). Table 12 indicates that longer hydrolysis times lead to higher umami taste values, with significant increases at each time interval (*p* < 0.05). The optimal treatment is 5 h of hydrolysis, which produces the highest umami taste value. Hydrolysis duration significantly affects the umami taste in the resulting hydrolysate. Other research demonstrated that longer hydrolysis duration produces shorter peptide bonds, enhancing umami taste (Fajriyah and Winarti 2022). Similarly, other research reported that extended hydrolysis time can increase glutamic acid content, which contributes to umami flavor (Wicaksono and Winarti 2021). Longer hydrolysis duration produces natural flavor enhancers with optimal umami taste intensity (Rosida 2022).

**TABLE 12:** Correlation between hydrolysis duration and glms umami taste value.

Time (h)	gLMS umami taste (mm)
2 h	33.14^a^
3.5 h	42.47^b^
5 h	48.82^c^
*F*‐value	543.412
df	2
*p*‐value	< 0.05

*Note:* Data were analyzed using Duncan's multiple range test; Different superscript letters indicate significant differences (*p* < 0.05).

#### 3.3.7 Effect of Degree of Hydrolysis on Umami Taste

Research data shows a correlation between hydrolysis duration and umami taste. Table 5 demonstrates that higher DH values correspond to increased gLMS values, indicating that increased hydrolysis degree contributes to umami flavor development. Other Research found that higher hydrolysis degrees directly correlate with increased free amino acid content, particularly glutamate, which contributes to umami taste (Haryati et al. 2010). High DH values in shrimp processing byproducts generate umami‐contributing compounds (Fajriyah and Winarti 2022). Optimal umami taste in mulberry leaf and shrimp head hydrolysates with the highest DH (84.82%) (Kartikosari 2024). More complex umami flavors in catfish protein hydrolysis products with high DH values (Rosida and Priyanto 2024). Enzymatic hydrolysis of snail protein with high DH values enhanced umami taste through free amino acid formation (Rhyu and Kim 2011). When molecular weight drops below 1 kDa, free amino acids are released from peptide terminals, enhancing umami taste (Lioe et al. 2007). Peptides under 500 Da significantly contribute to umami perception (Großmann et al. 2020). Previous research confirmed that ProteAX hydrolysis significantly intensifies umami taste by releasing substantial free amino acids, especially glutamic and aspartic acids. This enzymatic process effectively transforms cricket protein into a more palatable, flavor‐enhanced ingredient (Perez‐Fajardo et al. 2023).

### 3.4 Techno‐Functional Characteristics

#### 3.4.1 Solubility

The hydrolysate obtained under the condition of 1.5% enzyme‐to‐substrate ratio and 5 h hydrolysis time (sample XI), which showed the highest DH (67.71%), was selected for subsequent functional. Various studies have shown that the low solubility of crickets poses a challenge. Cricket flour reduces gluten formation in products like pasta and bread, leading to a decrease in elasticity and texture changes (Bresciani et al. 2022). There is also a reduction in emulsifying activity and stability, which are crucial for creating a homogeneous mixture in baked goods (Ho et al. 2022). On the other hand, sausage formulations indicate that the low solubility of cricket flour causes undesirable texture changes, such as increased hardness and reduced moisture retention (Eggink et al. 2022). The low solubility of cricket flour remains a key challenge in maintaining the desired quality in various food products, highlighting the need for modifications to enhance its solubility and improve its functional properties.

Based on Table 13, the CP sample has a lower solubility percentage compared to CPH. This indicates an increase in solubility of up to 1.5 times due to the enzymatic hydrolysis process. Insoluble proteins contain non‐protein components such as chitin. Chitin is a complex polysaccharide found in the exoskeleton of crickets that is difficult to dissolve in water. Chitin contains β‐(1,4)‐N‐acetyl glucosamine bonds, which are hard to break down by digestive enzymes, potentially reducing protein digestibility and acting as an antinutrient (Grossmann et al. 2021).

**TABLE 13:** Solubility test results.

Sample	Average solubility (%)
CP	65.95 ± 0.72
CPH	98.99 ± 0.55

*Note:* Data is presented as mean ± standard deviation (*n* = 2).

Abbreviations: CP, cricket protein; CPH, cricket protein hydrolysate.

Enzymatic hydrolysis significantly improved cricket protein solubility, with the hydrolyzed sample (CPH) demonstrating an exceptional 98.99% solubility. This enhancement results from reduced molecular weight and increased molecular entropy or movement freedom (Purschke et al. 2018). Protein hydrolysis degree (DH) critically influences solubility, with the CPH sample with 67.71% DH exhibiting superior solubility compared to the CP sample's 23.42% DH. This finding aligns with other research that found that protease application significantly increases DH, thereby improving the solubility and functional properties of insect protein (Guo et al. 2012). During enzymatic hydrolysis, large protein molecules are broken down into smaller peptides, increasing molecular mobility and water interaction. Conversely, low degrees of hydrolysis may promote protein–protein interactions, potentially leading to aggregation and reduced solubility (Purschke et al. 2018).

#### 3.4.2 Emulsification

Emulsification capability testing was conducted by storing samples at acidic, basic, and neutral pH levels, along with varying storage durations. This approach allows for evaluating emulsion stability and characterizing property changes under different environmental conditions. Emulsion testing at different storage times provides insights into the emulsion's shelf life and the duration a product can maintain its structural integrity before significant alterations occur. The pH conditions of the solution offer a comprehensive understanding of interactions between oil droplets and emulsion matrix under acidic, basic, and neutral environments. pH changes significantly influence protein emulsion stability and characteristics. pH conditions can affect protein secondary structures, thereby enhancing absorption capacity at the water–oil interface in emulsions (Chen et al. 2018). Higher pH alters the surface charge of emulsifiers and interactions between dispersed and dispersing mediums (Veider et al. 2022). To evaluate emulsion stability and characteristics under neutral conditions, samples were stored at pH 7, which represents a standard condition similar to human body pH and prevalent in many food products (standard physiological and food processing reference) (Zhao et al. 2019; McClements 2015). This pH serves as a critical benchmark for assessing protein hydrolysate performance across various food applications. The emulsion property test results for CP and CPH samples can be observed in the following table.

Based on Table 14 CP samples showed emulsion instability across all three pH conditions, demonstrated by an increase in droplet concentration along with increasing droplet size in increasingly irregular shapes. Under acidic conditions (pH 4), there was a rapid increase in droplet concentration each day, but with more uniform and stable shapes compared to neutral (pH 7) and basic (pH 10) conditions, indicating that CP samples were more stable in acidic conditions. The higher number of droplets in the emulsion can be one cause of low emulsion stability. According to other research, the increase in droplet concentration occurs due to reduced droplet viscosity or changes in surface charge distribution, which facilitates fragmentation into smaller and more numerous particles (Al Awwaly et al. 2017). Furthermore, weak stability properties can accelerate coalescence, causing droplets to break and merge, thus disrupting emulsion homogeneity. High droplet concentration expands the interface area, increasing inter‐droplet interactions and the probability of coalescence or the event of globule or droplet merger (Suprobo and Rahmi 2015). This coalescence phenomenon was experienced by CP samples under basic conditions (pH 10) and was more severe under neutral conditions (pH 7). Under both conditions, coalescence occurred, causing droplet aggregation (increased droplet size) that became increasingly severe and even increased the likelihood of demulsification or separation of the oil phase and sample solution into two immiscible liquids. Under basic conditions (pH 10), the initial droplet size was larger with irregular shapes, but the droplet concentration was lower compared to neutral conditions (pH 7), indicating that CP samples were more stable under basic conditions (pH 10) than neutral conditions (pH 7).

**TABLE 14:** Emulsification properties cricket protein sample at pH 4, pH 7 and pH 10.

	Day‐0	Day‐1	Day‐2
pH 4	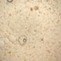	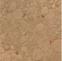	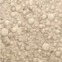
pH 7	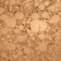	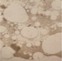	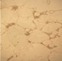
pH 10	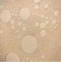	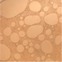	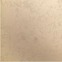

The coalescence phenomenon is a characteristic of droplet instability that occurs due to the concentration of interfacial film between droplet surfaces, causing the boundaries between merging droplets to disappear. This is followed by aggregation or formation of single droplets that have larger and non‐uniform sizes (Hernandez‐Rodriguez et al. 2024). The occurrence of coalescence and aggregation in most droplets can increase the likelihood of demulsification, causing the emulsion to become irreparable even through agitation (Mehrnia et al. 2016).

Based on Table 15, overall CPH samples demonstrated better emulsion stability, evident from smaller and more stable droplet sizes across all three conditions. Smaller droplet size indicates better stability (do Evangelho et al. 2017). This aligns with another study, which found that the formation of small globules in emulsions indicates the best emulsifying properties and protein emulsion stability (Song et al. 2022). Smaller oil phase particle sizes enable more uniform dispersion in the dispersing medium, indicating better structural organization and superior emulsion characteristics (Hernandez‐Rodriguez et al. 2024). Under acidic conditions (pH 7), droplet concentration was higher compared to other conditions, while under neutral (pH 7) and basic conditions (pH 10), both showed large droplet sizes on the first day but underwent breakage into smaller droplets on subsequent days and demonstrated better droplet stability. This indicates that CPH samples can remain stable in acidic, basic, and neutral conditions.

**TABLE 15:** Emulsification properties cricket protein hydrolysate sample at pH 4, pH 7 and pH 10.

	Day‐0	Day‐1	Day‐2
pH 4	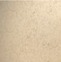	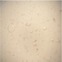	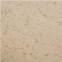
pH 7	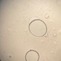	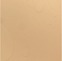	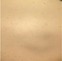
pH 10	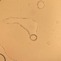	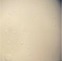	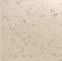

CPH samples demonstrated an emulsification process opposite that of CP samples. Under all three conditions, droplets that were initially large on day 0 gradually fragmented into smaller, dispersed droplets, indicating effective emulsification. This process occurs when emulsion stability works efficiently to maintain dispersed droplets in smaller sizes. Good emulsion properties can prevent oil droplet coalescence, thereby increasing mechanical strength and protecting nutrients from degradation (Erfando et al. 2019). The more stable an emulsion, the more difficult the demulsification process becomes in the mixture (Emuchay et al. 2013). This is due to the nature of emulsions as heterogeneous mixtures that allow liquids to be well dispersed in the form of droplets, making it difficult to separate the two phases in the mixture (Citrawan 2019; da Silva Lucas et al. 2020).

The emulsification capacity of cricket protein hydrolysates (CPH) demonstrates superior performance across acidic, basic, and neutral conditions compared to cricket protein (CP), characterized by smaller droplet sizes and minimal coalescence. Positive ions in acidic environments cause protein surface charge increases, enhancing inter‐particle repulsion and emulsion stability (Rayner and Dejmek 2015). Emulsion stability across varied pH conditions is crucial in food industries, particularly during storage processes exposed to diverse environments. Emulsification enables uniform substance distribution, improving texture, extending shelf life, and enhancing nutritional value in products like dairy, sauces, and dressings (Choi et al. 2017). Other research confirms that cricket protein emulsion properties significantly influence product texture, elasticity, and adhesiveness, necessitating careful formulation adjustments to maintain desired volume and structural characteristics (Ho et al. 2022; Ayu et al. 2021).

#### 3.4.3 Water Absorption Capacity

Water absorption capacity refers to a material's ability to absorb water to its maximum, which is crucial in determining the consistency, texture, and final quality of a product, including organoleptic and physical properties such as crispness and tenderness (Giescha et al. 2012). Therefore, water absorption capacity plays an important role in the functional properties of cricket flour. The observation was carried out by adding water to the CP and CPH samples, followed by centrifugation and weighing. The results of the water absorption capacity test for the CP and CPH samples can be seen in the following table:

Based on Table 16, CPH samples have a water absorption capacity up to 2.79 times higher than CP samples. The low water absorption in CP samples can be influenced by various factors. Cricket flour contains higher chitin which is difficult to absorb water due to its hydrophobic and insoluble nature, thus inhibiting CP's ability to absorb and retain water (Ashaolu 2020). This low absorption tends to produce harder end products. Other studies showed that low water absorption in cereal results in harder and less appealing texture (Giescha et al. 2012). Low absorption capacity results in analog rice products becoming hard and less palatable (Mugova et al. 2021).

**TABLE 16:** Water absorption capacity test results.

Sample	WAC (g/g)	WAC (%)	Average (%)
CP1	1.35	134.64	132.63 ± 2.84
CP2	1.31	130.62	
CPH1	3.71	371.36	370.46 ± 1.26
CPH2	3.69	369.57	

*Note:* Data is presented as mean ± standard deviation (*n* = 2).

Abbreviations: CP, cricket protein; CPH, cricket protein hydrolysate.

CPH samples can absorb water up to 370.46%. The high‐water absorption capacity in hydrolysates makes it potential as a formulation ingredient (Kim et al. 2017). High water absorption capacity significantly influences organoleptic quality across various food formulations (Mafu et al. 2022). Multiple studies highlight its critical role in food product development. Increased water absorption in cricket flour provides improved cake and bread texture with more stable dough structures. Enhanced water interaction is crucial for meat emulsion formulations, indicating better gel stability and reduced water loss during cooking (Bawa et al. 2020). Other research revealed that high water absorption cricket flour produces softer bread, increasing consumer preference (da Rosa Machado and Thys 2019). Other study confirmed that such formulations maintain product moisture during storage and enhance taste (Simeon et al. 2022). High water absorption cricket flour in gluten‐free bread creates stable, soft textures while extending shelf life without structural degradation (Yi et al. 2013). Suitability for breakfast cereal products requiring rapid hydration. These studies collectively underscore the transformative potential of water absorption capacity in cricket flour across diverse food applications.

#### 3.4.4 Oil Absorption Capacity

Oil absorption capacity refers to a material's ability to retain and absorb oil within its matrix, which is related to the hydrophobic properties of the protein. Oil absorption capacity is an indicator of a material's ability to maintain the desired texture and flavor in the final product (Kowalczewski et al. 2019). The oil absorption capacity was observed by adding oil to the CP and CPH samples, followed by centrifugation and weighing. The results of the oil absorption capacity test for the CP and CPH samples can be seen in the following table.

Based on Table 17, the CPH sample has an oil absorption capacity that is 2.96 times higher than the CP sample. The CPH sample is capable of absorbing up to 249.35% of oil. This means that for every 1 g of the sample, it can absorb up to 2.49 g of oil. The high oil absorption capacity of CPH indicates that the enzymatic hydrolysis process can break down proteins into smaller peptides, increasing the surface area that can interact with oil molecules. Hydrolysis process can increase the surface area of peptides, allowing for higher oil absorption (Hilkias et al. 2017).

**TABLE 17:** Oil absorption capacity test results.

Sample	OAC (g/g)	OAC (%)	Average (%)
CP1	0.86	85.73	84.17 ± 2.21
CP2	0.83	82.61	
CPH1	2.49	248.78	249.35 ± 0.81
CPH2	2.50	249.92	

*Note:* Data is presented as mean ± standard deviation (*n* = 3).

Abbreviations: CP, cricket protein; CPH, cricket protein hydrolysate.

Higher oil absorption capacity in cricket protein hydrolysate (CPH) compared to native cricket protein (CP) stems from structural changes and enhanced functional properties, including increased surface area, hydrophobic group exposure, and improved emulsification capabilities. Increased hydrolysate solubility optimizes peptide‐oil interactions, enabling more efficient oil molecule entrapment (Kowalczewski et al. 2019). The hydrolysis process renders peptides with superior emulsion abilities, creating protective layers around oil molecules and enhancing retention within protein matrices. Proteins with higher emulsion capacities better preserve oil molecules in protein networks. Other research confirmed that hydrolysis increases peptide side‐chain binding affinity with oil molecules, supporting enhanced oil absorption capacity. The simplified peptide structures resulting from hydrolysis facilitate improved interactions between peptide side chains and oil molecules, fundamentally transforming the protein's functional characteristics.

The enzymatic hydrolysis of cricket protein demonstrates interconnected improvements across multiple parameters, where higher Degree of Hydrolysis (DH) strongly correlates with enhanced sensory and functional properties. As DH increases from 6.85% to 50.34%–67.71%, there's a significant reduction in bitterness (32.88 to 5.92–16.16 mm gLMS) and bitter aftertaste, while simultaneously intensifying umami flavor (30.32 to 52.16 mm gLMS), suggesting that protein breakdown effectively reduces bitter compounds while generating desirable umami‐contributing peptides. The optimal processing conditions of 1.5% enzyme‐to‐substrate ratio (E/S) and 5‐h hydrolysis duration not only maximize these sensory improvements but also enhance functional properties, with Cricket Protein Hydrolysate (CPH) showing 1.5 times higher solubility, 2.79 times greater water absorption capacity, and 2.96 times increased oil absorption capacity compared to unhydrolyzed Cricket Protein (CP), along with improved emulsion stability characterized by smaller and more stable droplets. These improvements are interconnected, as increased solubility may contribute to better taste perception through greater compound availability to taste receptors, while also supporting enhanced emulsion properties and absorption capacities through better protein‐water–oil interactions, though pH sensitivity remains important for specific applications, with optimal emulsion stability observed at pH 4. This comprehensive enhancement across multiple parameters indicates that the enzymatic hydrolysis process creates a cascade of improvements through protein structure modification, simultaneously achieving better sensory properties and enhanced functional characteristics, making the hydrolyzed cricket protein more suitable for food applications.

## 4 Conclusion

The study revealed that enzymatic hydrolysis of crickets ( *Acheta domesticus* ) using ProteAX enzyme at the best enzyme‐to‐substrate ratio of 1.5% over 5 h significantly improved protein characteristics. This condition yielded the highest degree of hydrolysis (67.71%) with remarkable organoleptic properties, characterized by low bitter taste (5.9 mm), low aftertaste (2.8 mm) and a strong umami taste (52.16 mm), while successfully increasing protein content to 79.51% on a dry basis. The statistical analysis shows that enzyme concentration and hydrolysis time independently affect DH and bitter taste, with no significant interaction, while aftertaste and umami taste show an interaction. Moreover, hydrolysis demonstrated substantial enhancements in techno‐functional qualities, including a 1.5‐fold increase in solubility (98.99%), a 2.79‐fold improvement in water absorption capacity (370.46%), and a 2.96‐fold enhancement in oil absorption capacity (249.35%). Furthermore, cricket protein hydrolysate exhibited superior emulsion stability across various pH conditions, highlighting its potential as an innovative and versatile ingredient in advanced food formulation strategies.

## 5 Recommendations

Further studies are needed to optimize enzymatic hydrolysis conditions with ProteAX to achieve better results. Additional testing on the functional properties of cricket protein hydrolysates is also required to understand their potential benefits in various industrial applications.

## Author Contributions

**Fetriyuna Fetriyuna:** conceptualization, methodology, supervision, writing – review and editing, validation, investigation, data curation. **Nandi Sukri:** conceptualization, supervision, methodology. **Ratna Chrismiari Purwestri:** validation, formal analysis, writing – review and editing. **Rozanah Nushrotina:** writing – original draft, writing – review and editing, formal analysis, data curation, funding acquisition, project administration. **Ade Chandra Iwansyah:** funding acquisition, methodology, validation, writing – review and editing, supervision, project administration.

## Funding

This work was supported by Badan Riset dan Inovasi Nasional.

## Conflicts of Interest

The authors declare no conflicts of interest.

## Data Availability

The data that support the findings of this study are available from the corresponding author upon reasonable request.
